# NMDAR autoantibodies in psychiatric disease - An immunopsychiatric continuum and potential predisposition for disease pathogenesis

**DOI:** 10.1016/j.jtauto.2022.100165

**Published:** 2022-09-21

**Authors:** Niels Hansen

**Affiliations:** a Department of Psychiatry and Psychotherapy, University Medical Center Göttingen, Göttingen, Germany; b Translational Psychoneuroscience, University Medical Center Göttingen, Göttingen, Germany

**Keywords:** Immunopsychiatry, NMDAR antibody, Autoimmune encephalitis

## Abstract

*N*-Methyl-d-Aspartate-receptor (NMDAR) antibody encephalitis is a disease discovered two decades ago. Our knowledge about it has recently deepened dramatically. However, the significance of NMDAR antibodies in psychiatric disease cannot be determined if there are no clear indications of brain inflammation or an autoimmune encephalitis mediated by NMDAR antibodies. Furthermore, the long-term interaction and connection between these two disease entities are unclear. In this paper we aim to elucidate the relationship between these disease entities. We propose two distinct models that explain the on the one hand a condition in which a minor inflammatory state as in psychiatric disease culminates in a severe state of inflammation characterized by NMDAR encephalitis. On the other hand, we postulate a model in which an NMDAR encephalitis might later create favorable conditions for inducing psychiatric disease. These models should be kept in mind for further investigations examining the long-term outcome of NMDAR autoantibody immunity in the brain and its functions.

## 1 NMDAR autoantibodies in psychiatry

*N*-methyl-D-aspartate (NMDAR) autoantibodies have attracted growing research interest over the last two decades. NMDAR autoantibody mediated encephalitis (NMDARE) is an independent disease entity possessing psychiatric and neurological clinical features [[1], [2], [3]]. Probable NMDAR encephalitis can be diagnosed if four of six specific clinical features are fulfilled, ie, the presence of aberrant psychiatric behavior, speech dysfunction such as mutism, abnormal movements such as catatonia, disturbances of consciousness, seizures or autonomic dysregulation [4] in addition to proven NMDAR autoantibodies in serum and/or cerebrospinal fluid (CSF). Furthermore, one of two laboratory criteria must also be met to diagnose NMDARE: either a pleocytosis in CSF or electroencephalographic (EEG) abnormalities such as focal or diffuse slowing, epileptic potentials, or an extreme delta brush pattern [4]. The Graus criteria [4] classify NMDARE as probable if a systemic teratoma is present and only three of four of the aforementioned clinical criteria are fulfilled. However, an NMDARE is proven if just one of the clinical features is present and CSF immunoglobulin G (IgG) NMDAR N1 antibodies are detected, according to the Graus criteria [4]. The onset of NMDARE is often characterized by psychiatric symptoms [5,6]. Nevertheless, NMDAR autoantibodies are also detected in psychiatric patients who fail to fulfill NMDAR-encephalitis criteria [[7], [8], [9]]. The role NMDAR autoantibodies play in these patients is less well understood, and deserves investigation in large-scale studies. Both a low-grade inflammation like a mild encephalitis [10] or neuroprotective NMDAR antibodies are discussed [11]. The type of NMDAR autoantibodies is highly relevant. NMDA receptors are composed of heterotetramers which in turn have glutamate GluN1 (NR1) and GluN2/3 (NR2) subunits. Emerging evidence suggests that NMDAR GluN2 (NR2) autoantibodies and NMDAR autoantibodies of the immunoglobulin A (IgA) or immunoglobulin M (IgM) type are probably less pathogenic in a clinicopathological presentation like encephalitis [12]. IgG-subtype-NMDAR antibodies are usually identified in patients with autoimmune encephalitis, while NMDAR antibodies of the IgA or IgM subtype are more likely to be present in atypic manifestations of progressive or unclassifiable dementia [[12], [13], [14]]. In addition, IgG-type NMDAR antibodies trigger detectable changes in NMDAR at the receptor level, whereas that fails to occur with IgA- or IgM-subtype NMDAR antibodies [12]. Animal studies recently demonstrated the redistribution of NMDAR receptors on the membrane surface after exposure to NMDAR autoantibodies against the GluN1 subunit (NR1), and thus the pathogenicity of NMDAR NR1 antibodies [15]. In contrast, NMDAR NR2 antibodies were shown to be associated with a neurobehavioral phenotype in a mouse animal model, thus also being potentially pathogenic [16]. Interestingly, NMDAR NR1 antibodies are produced under circumstances as varied as stress, cancer, infection, or brain injury to act as endogenous NMDAR antagonists, thus exhibiting both deleterious and beneficial effects [17]. In a study using sera containing NMDAR NR1 autoantibodies from different disease conditions, neurons from pluripotent stem cells were shown to internalize NMDAR [18]. Furthermore, in Xenopus oocytes, these NMDAR NR1 antibody-containing sera were shown to lead to weaker glutamate-signaling currents [18]. NMDAR NR1 autoantibodies are thus potentially pathogenic. The NMDAR NR1 subtype plays an essential role in synaptic signaling in the brain by regulating intracellular Ca2+ levels [19], although NMDAR NR2 (GluN2A) antibodies are also important for synaptic plasticity through their related intracellular signal cascades [20]. The NMDAR NR1 antibody is the most studied antibody subtype. Nevertheless, cell-based assays have also examined the GluN2B subunit in addition to NMDAR NR1 [21]. Most commercially available antibody screening tests employ monotransfected human embryonic kidney cells in the form of a biochip test for NMDAR NR1 [21]. In addition, note that the GluN1 subunits of NMDAR autoantibodies are usually detected in NMDAR-positive encephalitides, whereas the GluN2 subunits of NMDAR antibodies are associated with lupus erythematosus [22]. Our narrative review describes the most recent developments in the pathogenesis in psychiatric syndromes and disorders associated with anti-NMDAR antibodies.

## 2 NMDAR autoantibodies in psychiatric autoimmune encephalitis

Psychiatric symptoms like psychotic or depressive symptoms often characterize the onset of NMDARE [23,24]. Psychiatric symptoms frequently culminate in cognitive dysfunction at a later stage of NMDARE [25]. However, the pure manifestation of psychiatric symptoms in NMDARE seems to be rare, as shown in a recent investigation by Kayser et al. [3]. NMDARE is often characterized by severe inflammation in the central nervous system (CNS) when compared to other types of membrane-surface autoantibody-related encephalitis types such as LGI1 encephalitis proven by frequent CSF abnormalities [26] such as pleocytosis [27]. Furthermore, oligoclonal bands (OCBs) are often found accompanying a severe functional impairment in NMDARE [26]. Thus, NMDARE is a prototype of a relative extended neuroinflammation unlike other types of autoimmune encephalitis such as glycine or LGI1 associated encephalitis as shown in a recent CSF study from Blinder and Lewerenz [28]. It is essential to examine autoantibodies against NMDAR NR1 in CSF as well as serum, because their presence in CSF with typical clinical features supports the diagnosis of definite autoimmune encephalitis according to the Graus criteria [4]. In addition, a recent study by Blackman [9] demonstrated that psychiatric syndromes associated with NMDAR antibodies can differ from psychiatric syndromes associated with serum NMDAR antibodies on the basis of clinical demographic factors and diagnostic features. For example, patients presenting CSF NMDAR antibodies unlike those with serum NMDAR antibodies) were more likely to suffer a subacute onset, more likely to be female, more likely to present EEG and CSF abnormalities, and more likely to be psychotic and suffer from insomnia [9]. A very recent study also demonstrated that patients in the post-acute stage of NMDAR encephalitis resemble in their cognitive abilities those with stabilized schizophrenia [29]. These studies show the importance of making a differentiated diagnosis via CSF and serum autoantibody assessments, and of documenting clinical features like positive symptoms such as psychosis, but also cognitive performance in both the acute and post-acute stage.

## 3 Pathophysiological role of NMDAR autoantibodies in psychiatric NMDARE

Recent animal studies [30] demonstrated that NMDAR-1 antibodies suffice to induce and modulate psychiatric behavior in mice brains presenting brain inflammation caused by genetic abnormalities before exposure to NMDAR antibodies. NMDAR autoantibodies might therefore be relevant in inducing psychiatric symptomatology in humans, but they might be not caused an NMDAR encephalitis involving brain inflammation. More research in humans is needed to pursue this observation originating from animal studies. Another critical issue is the temporal exposure of NMDAR in the brain. Another animal study showed that the chronic, but not acute circulation of low titer NMDAR1 autoantibodies in blood led to impaired spatial working memory and novelty detection in mice possessing an intact blood brain barrier function [31]. These results are extremely important to explaining why NMDAR might still be pathogenic despite the lack of clear indices of an NMDARE. Another research group however showed that learning and memory (via Water maze testing) were unaffected, but mice exposed to NMDAR1 autoantibodies displayed psychosis-like behavior and loss of inhibition [32]. These studies indicate the relevance of NMDAR1 autoantibodies in modulating psychiatric behavior in the long run, but dampen the significance of NMDAR1 autoantibodies in inducing encephalitis. Nevertheless, it has to be kept in mind that it is very difficult to compare behavioral manifestations in animals with psychiatric and cognitive symptoms in humans. Thus, more research is needed to characterize the pathogenic role of NMDAR autoantibodies in humans [33] such as a role in generating psychotic symptoms [34,35].

## 4 NMDAR autoantibodies in psychiatric disorders

**Fig. 1:**
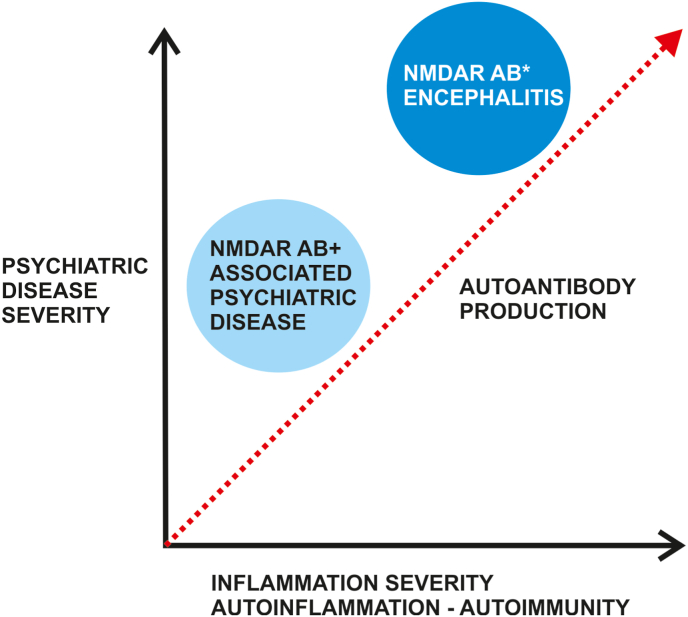
A proposed model for NMDAR antibody continuum between psychiatric disease associated with NMDAR antibodies and NMDAR encephalitis We propose that low NMDAR autoantibody status associated with psychiatric disease might predispose conditions for later development of NMDAR encephalitis if autoantibody production is triggered by autoinflammation that itself might be induced by specific tumor or viruses. The psychiatric symptom severity is enhanced if NMDAR encephalitis is evolved. Abbreviations: AB+ = autoantibody positivity, NMDAR = *N*-methyl-D-aspartate receptor.

A large investigation of 1661 patients [36] over 12 years revealed that NMDAR-autoantibody seropositivity is low (3.8%). Many observations have suggested that psychotic symptoms deserve more examination entailing the search for autoantibodies if these appear suddenly and last a short time. NMDAR autoantibodies were tested to detect NMDAR antibodies even before the development of an acute psychosis in a medium-sized cohort of 254 patients presenting a high clinical risk for psychosis. However, the frequency of NMDAR antibodies did not differ significantly between patients carrying a high risk for psychosis and controls (8.3% vs. 5.2%) [37]. Nevertheless, small changes in psychopathology were observed between groups. Patients carrying a high risk for psychosis and NMDAR antibodies exhibited higher levels of depressed mood, dysfunctional verbal learning, and more pronounced cognitive dysfunction than NMDAR antibody-positive controls [37]. These results indicate that the NMDAR autoantibodies in psychotic patients might reveal another pathogenicity than those in controls even when the NMDAR-antibody status does not differ. Furthermore, this may be evidence of the onset of an autoimmune continuum (Fig. 1) involving a mild encephalitis developing into a severe autoimmune encephalitis fulfilling the latest NMDARE criteria. A systematic review of NMDAR antibodies in psychiatric disorders pointed out that the seropositivity varies widely, ranging from 0.5 to 11.6% depending on the study population and testing methods (fixed or live cell-based assay) [38]. These authors suggested that the NMDAR autoantibodies play a role in a subgroup of psychotic patients [38]. A very recent investigation highlighted a specific psychopathology that may indicate an NMDARE, such as a polymorphic psychotic symptomatology, a fluctuating course with marked cognitive dysfunction and psychotic symptoms [39]. Thus, the psychopathology in relationship to the NMDAR-autoantibody status must be investigated in more depth in future studies.

## 5 Pathogenic role of NMDAR antibodies in psychiatric disorders

**Fig. 2:**
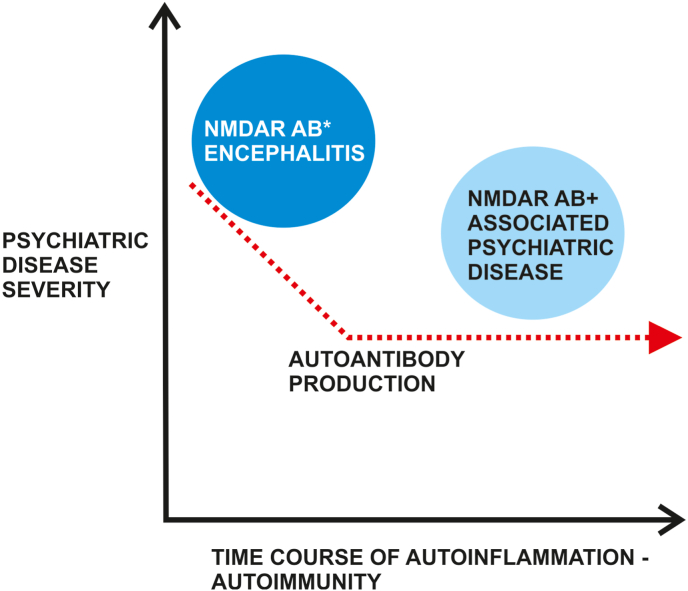
A putative model for an NMDAR antibody continuum between NMDAR encephalitis and the later induction of psychiatric disease associated with NMDAR antibodies Abbreviations: AB+ = autoantibody positivity, NMDAR = *N*-methyl-D-aspartate receptor. A NMDAR encephalitis might predispose the brain's conditions to develop at a later stage a psychiatric disease either associated with NMDAR autoantibodies or not. The autoinflammation and clinical severity drop from an initial high stage to a moderate stage.

NMDAR antibodies might be relevant in inducing psychiatric behavior if they circulate over a long time period, as evident in animal studies. A recent report showed that schizophrenia occurred after an NMDARE [40], tempting us to postulate a relevant pathogenic role of encephalitis involving NMDAR antibodies in that patient's schizophrenia development. The NMDAR dysfunction induced by NMDAR autoantibodies might lower the threshold for psychosis, and the threshold for a schizophrenia might be overcome by a long-term lowered threshold for psychosis, thus predisposing for psychiatric disease (Fig. 2). It seems relevant if neurodegeneration markers such as neurofilament light chains are elevated and exceed 15 pg/ml in patients experiencing their first psychotic episode, if those patients develop NMDARE [41]. Another issue seems to be the presence of CSF-NMDAR antibodies, as such CSF-NMDAR autoantibodies are associated with a higher percentage of patients suffering from psychiatric symptoms [42]. A recent investigation demonstrated that patients displaying psychotic symptoms are not the most frequent patients presenting NMDAR antibodies, as they were most frequently detected in conjunction with bipolar disorder (10%) and less often but still frequently identified accompanying schizophrenia (2.4%) or depression (3%) [43]. An investigation by Pearlman and Najjar [44] delivered similar results, demonstrating that schizophrenia, schizoaffective disorder, bipolar or major depressive disorder are associated with a three-times higher risk of having elevated NMDAR antibody titers than healthy controls. The risk of a psychosis was 11-fold higher in patients whose NMDAR-NR2 antibodies concentrations were lower than 2.92 ng/ml [45]. In addition to the type of psychiatric disease, the mechanistic basis of NMDAR pathology seems highly relevant for the phenotype. An animal study proved that NMDAR trafficking from extracellular to intracellular sites could be responsible for the development of psychosis [46], so that this mechanism should be considered when studying the disease pathogenesis. CSF antibodies from schizophrenic patients can induce just such NMDAR trafficking [46]. These animal studies therefore provide a basis showing how psychotic symptoms might be induced. Further studies are needed to see if a switch in NMDAR dynamics can be induced by NMDARE that paves the way for lowering the threshold for developing psychosis or schizophrenia via altered NMDAR dynamics (Fig. 2). A recent study indicated an NMDAR-dependent mechanism affecting the integrity of the white-matter microstructure in schizophrenia [47]. Thus, white matter-structural integrity as a result of NMDAR autoimmunity could be the consequence that facilitates the development of psychiatric disease (Fig. 2). Note that methodological factors have been respected throughout all these theoretical reflections. The method of detecting autoantibodies plays a major role, as a live cell-based assay is recommended for a better estimation of the NMDAR-antibody prevalence [48], which seems particularly relevant in those patients in whom lower autoantibody titers would be likely, as in NMDAR autoantibody-associated psychiatric disease and not NMDARE itself (Fig. 1, Fig. 2). In such cases other tools might help identify patients with a potential NMDAR-antibody associated encephalitis, like quantitative EEGs with a fast-slow ratio index as a very sensitive early sign of NMDAR encephalitis [49]. There are other markers of neuronal damage in the same context that might help identify first-episode psychosis patients with NMDAR antibodies as neurofilament light chains is a particular marker of disease severity in first-episode psychosis, but not a long-term disease marker, as a recent study by Guasp et al. [41] showed. Old and novel biomarkers could thus help us interrupt the autoimmune continuum of NMDAR pathogenicity from psychiatric diseases associated with NMDAR autoantibodies and NMDARE (Fig. 1, Fig. 2).

## 6 Discussion

Although much research has been conducted explaining how NMDAR antibody-mediated autoimmunity might contribute to psychosis, much less is known about the generation of bipolar symptoms. We propose a continuum of autoimmunity between psychiatric disorders, especially psychosis or bipolar disorder associated with NMDAR antibodies, and definitive autoimmune encephalitis with NMDAR antibodies (Fig. 1, Fig. 2). We postulate two different conditions framed in two models that interact dynamically (Fig. 1, Fig. 2). On the one hand, NMDAR encephalitis might be predisposed by a schizophrenic condition, as exemplified in a recent case report [50] (Fig. 1) or NMDAR encephalitis can predispose the conditions for developing a psychiatric disease (Fig. 2) such as schizophrenia in mice [51] and humans [40]. In other words, the type of inflammation can culminate in an autoimmune condition if the inflammation is severe enough to prime the individuum for set point modulation resulting in a psychiatric disease (Fig. 1). On the other hand, the inflammation's severity may weaken, leading to a mild inflammation bearing the conditions for inducing psychiatric disease as well (Fig. 2). A severe inflammation is proposed to be associated with NMDAR encephalitis, whereas a mild inflammation possibly contributes to chronic psychiatric disorder such as schizophrenia or chronic bipolar depressive disorder. We do not know what type of environmental factors (viral or tumor-related) can trigger the set point for the developing a state of NMDAR antibody inflammation severe to mild (Fig. 2), or mild to severe (Fig. 1)]. Quantifying the encephalitis should be a major aim to identify those patients who only present a mild form of encephalitis but who could also benefit from immunotherapy. However, first-episode psychoses with and without NMDAR antibodies have so far not differed in their response to antipsychotic drugs [37], making a therapeutic distinction between these entities difficult. The mechanisms of NMDAR antibody for disease pathophysiology (gating of NMDAR or altering of the synaptic components of NMDAR) might be important for the psychiatric phenotype that results. Moreover, studies addressing the long-term consequences of NMDAR antibodies on brain morphology and function due to autoinflammation would be worthwhile to clarify the role NMDAR antibodies play in inducing and maintaining psychiatric disease. Local brain microcircuits in specific brain regions such as the prefrontal cortex might be disturbed by NMDAR antibodies involving failing synaptic transmission, resulting in altered long-term potentiation [52]. These mechanistic actions can trigger symptoms such as working memory deficits in psychiatric disease like schizophrenia.

## 7 Synopsis

Our findings might suggest that an autoimmunity continuum exists that ranges from NMDAR antibodies associated with psychiatric disease to probable to definitive NMDARE (Fig. 1). Thus, we postulate the following model depicted in Fig. 1: the psychiatric disease severity worsens when the autoinflammation increases and autoimmunity phenomena develop along with rising production of NMDAR antibodies. Two main phenomena can be distinguished such as psychiatric disease associated with NMDAR antibodies, and NMDAR antibody-mediated encephalitis. However, this model does not consider an initial attack on the brain induced by NMDAR encephalitis that later develops to an NMDAR antibody-associated chronic psychiatric disease like schizophrenia. Thus, we developed a second model (Fig. 2) that integrates an initial severe inflammation attack on the brain entailing a chronic psychiatric disease that appears later. Furthermore, there is evidence that an initial NMDARE might initiate an autoimmune attack that predisposes the brain to develop a psychiatric disease like schizophrenia later. Such a strong initial attack in the form of an NMDARE might be detected by specific brain morphology such as atrophy, ie, cerebellar atrophy [53].

## Author contributions

NH wrote the manuscript.

## Funding

This study was funded by the Open Access fund of the University of Göttingen.

## Declaration of competing interest

The author declares that he has no known competing financial interests or personal relationships that could have appeared to influence the work reported in this paper.

## Data Availability

No data was used for the research described in the article.
